# The Effect of Adding the Leaves and Fruits of *Morus alba* to Rape Honey on Its Antioxidant Properties, Polyphenolic Profile, and Amylase Activity

**DOI:** 10.3390/molecules25010084

**Published:** 2019-12-24

**Authors:** Monika Tomczyk, Michał Miłek, Ewelina Sidor, Ireneusz Kapusta, Wojciech Litwińczuk, Czesław Puchalski, Małgorzata Dżugan

**Affiliations:** 1 Department of Chemistry and Food Toxicology, Institute of Food Technology and Nutrition, University of Rzeszów, Ćwiklińskiej 1a St., 35-601 Rzeszów, Poland; mkkmilek@gmail.com (M.M.); ewelina.sidor@poczta.onet.pl (E.S.); mdzugan@ur.edu.pl (M.D.); 2 Department of Food Technology and Human Nutrition, Institute of Food Technology and Nutrition, University of Rzeszów, Zelwerowicza 4 St., 35-601 Rzeszów, Poland; ikapusta@ur.edu.pl; 3 Department of Plant Physiology and Biotechnology, Institute of Agricultural Sciences, Environment Protection and Management, University of Rzeszów, Ćwiklińskiej 1a St., 35-601 Rzeszów, Poland; wlitw@ur.edu.pl; 4 Department of Bioenergetics and Food Analysis, Institute of Food Technology and Nutrition, University of Rzeszów, Zelwerowicza 4 St., 35-601 Rzeszów, Poland; cpuchal@ur.edu.pl

**Keywords:** mulberry, honey, antioxidant activity, enzymatic activity, polyphenols, UPLC

## Abstract

Two various species of mulberry (*Morus* sp.) were selected to enrich rape honey with dried leaves or lyophilized fruits (4% *w*/*v*). Finally, fruits and leaves of the ‘Ukraińska’ clone were introduced into the honey during creaming in concentrations from 1 to 4% *w*/*v*. The total phenolic content, antioxidant activity, anthocyanins content, and polyphenolic profile were tested in plant extracts and enriched honeys. Moreover, α-glucosidase, β-galactosidase, and diastase activities were investigated in honeys. For mulberry extracts, chlorogenic acid isomers and rutin were considered main antioxidant compounds. The antioxidant activity of honey enriched with mulberry leaves increased even more than 50 times, due to introducing numerous phenolic acids and flavonoid glycosides. A significant decrease in the diastase activity in honey depending on the content of added mulberry leaves (almost 50% decrease in the case of 4% addition) was found, suggesting the inhibitory effect of honey with mulberry leaves against carbohydrate hydrolyzing enzymes.

## 1. Introduction

Mulberry (*Morus* sp., *Moraceae*) is widely distributed in the temperate, subtropical, or tropical regions of the world and can grow in a wide range of climatic, topographical, and soil conditions. There are 24 species of the *Morus* genus, and the most notable are white mulberry (*Morus alba*), black mulberry (*M. nigra*), and red mulberry (*M. rubra*) [1]. Being an excellent source of nutrients and phytochemicals, mulberry has been established as functional food [2]. In folk remedies, various parts of the mulberry tree, including root bark, leaves, and fruits, have been traditionally used for the treatment of fever, cough, hyperlipidaemia, hypertension, and hyperglycaemia [3]. However, in most European countries, mulberries are grown for fruit production rather than foliage. 

Mulberry fruit contains sugars, organic acids, free amino acids, vitamins, micronutrients, and other components [4]. Moreover, it has been found that mulberry fruit extract exhibits strong antioxidant properties, which are generally attributed to the presence of phenolic compounds, especially anthocyanins. However, mulberry fruits, even from the same species, can differ strongly regarding chemical composition, as well as antioxidant properties [5]. The fruits are usually consumed fresh but also dried, juiced, and canned, and jams and mulberry wines have been developed. In China, mulberry fruits have been used as important medicinal materials [6].

Mulberry leaves play a pivotal role in the sericulture industry because they serve as the sole food of the silkworm (*Bombyx mori*) [7]. Some reports indicate that mulberry leaves have a high content of bioactive compounds, including phenolic acids, flavonoids, alkaloids, and γ-aminobutyric acid (GABA). These compounds have been confirmed to exhibit antioxidant, antihypertensive, and anti-inflammatory effects, preventing atherosclerosis and decreasing glycemia [8]. One of the main bioactive compounds in mulberry leaves is 1-deoxynojirimycin (DNJ), a polyhydroxylated piperidine alkaloid that exhibits inhibitory properties with respect to α-glycosidases and shows potential therapeutic effects on many diseases, particularly type II diabetes [9]. Herbal tea made from mulberry leaves is consumed as a healthy beverage among Asian countries. Moreover, mulberry leaves-derived products in the form of powders, extracts, and capsules are now commercially available as functional foods and dietary supplements for controlling body weight and blood glucose [3].

Many varieties of mulberry are grown all over the world. They differ in morphology, shape, and color of the fruit. Four mulberry clones were used in this work: One variety of *Morus bombycis*, ‘Kenmochi’ with black fruits (length 1–2 cm), and three clones of *Morus alba*: ‘Ukraińska’ with light fruit with a light shade of pink (1–2 cm), ‘Bistro’ with short white fruits (1–1.5 cm), and ‘Żółwińska wielkolistna’ with slightly longer (1.5–2.5 cm) black fruits. Leaves of the variety ‘Żółwińska wielkolistna’ are larger than the others (up to 15 cm diameter). Mulberry exhibits heterophylly: On one tree, leaves of different shapes can be found, heart-shaped or lobed.

The nutritional and therapeutic value of honey has been acknowledged in the scientific literature; however, its use in metabolically compromised patients, including diabetics, remains controversial [10]. Some scientific opponents state that honey could not be used in a diabetic patient’s diet, due to the high content of carbohydrates, whereas supporters believe that fructose, the predominant sugar in honey, has a low glycemic index, advisable in the diabetic diet [11,12,13]. As the method of honey enrichment with herbs has been developed and the consumer interest of such products has recently grown [14], the introduction of mulberry fruits or leaves to bee honey seems to be a good idea. The combination of honey, a controversial ingredient for diabetics with a product that clearly reduces blood sugar level like mulberry, may be a modern healthy sweetener for patients suffering from diabetes.

The aim of this study was to investigate the effect of the addition of *Morus alba* leaves and fruits to rape honey during creaming on the antioxidant properties, phenolic profile, and activity of native glycoside hydrolases in enriched honey.

## 2. Results and Discussion

In the first step of the research, the comparison of polyphenols and anthocyanins content (in fruits only), as well as the antioxidant capacity, in leaf and fruit extracts from four mulberry cultivars was tested. The comparison included three clones of species *Morus alba*, as well as those belonging to the *Morus bombycis* ‘Kenmochi’ cultivar. The results confirming the occurrence of species- and cultivar-specific differences are presented in Table 1.

Among tested samples, the highest phenolics content and strongest radical scavenging activity (2,2-diphenyl-1-picrylhydrazyl (DPPH) test) and reducing power (ferric reducing ability of plasma (FRAP) assay) were found in the fruits of cultivars characterized by their black color (*M. alba* ‘Żółwińska wielkolistna’ and *M. bombycis* ‘Kenmochi’). The result was probably caused by the large content of anthocyanins in such fruits, which were determined in significantly higher amounts (*p* < 0.05) as compared to white fruits. In the case of these two samples, anthocyanins represent a significant majority of polyphenol compounds: 59 and 68.5%, respectively. In cultivars with white (‘Bistro’) or slightly pink fruits (’Ukraińska’), the anthocyanins account for 17 and 20.5% of total polyphenols, respectively. Moreover, the anthocyanins significantly affect the antioxidant capacity of fruit extracts, which was confirmed by the strong positive Pearson correlation coefficient (*r* = 0.990) (Table 2). Extracts of the slightly pink fruits showed negligible antioxidant activity compared to the black fruits. The observed differences are in agreement with literature data confirming that the polyphenol content is associated with the phenotype of the mulberry plant. Gungor and Sengul [15] determined the total phenolic content and antioxidant activity for three phenotypes of white mulberry fruits at an average level of about 19 μg GAE/mg (1900 mg GAE/100g). Dimitrova et al. [16] compared the fruits of three mulberry species: White, black, and red mulberry, and found the total content of polyphenols in the range from 400 mg GAE/100 g for *M. alba* to 900 mg GAE/100 g for *M. rubra*. The reducing power determined by FRAP assay in their study was much higher than that in our study (for *M. alba*, up to 80 mmol TE/100 g); however, the key issue here may be the method of extraction, especially the use of 70% ethanol as a solvent [16]. Black mulberry fruits were also tested for the DPPH scavenging effect by Kucelova et al. Data obtained by the authors indicate a high ability to inhibit the DPPH radical by fruit extracts (about 60–70%) [6]. However, they did not show the relationship between anthocyanin content and antiradical activity.

In the case of mulberry leaves, the highest polyphenol content was obtained for *M. alba* ’Ukraińska’ (761.4 mg GAE/100 g DW) and ‘Żółwińska wielkolistna’ (680.6 mg GAE/ 100 g DW), whereas for the other clones, significantly lower values were determined (*p* < 0.05). The greatest ability to scavenge the DPPH radical (63.5%) and reducing power (3.02 mmol TE/100 g) were found for ‘Ukraińska’ leaves. Literature data indicate varied content of polyphenols in mulberry leaves, depending on species, variety, harvest time, drying, and extraction conditions. In the research of Dimitrova et al. [16], the total phenolics content (TPC) values varied between 30 for *M. alba* and 220 mg GAE/100 g for *M. rubra*. Khyade obtained a value of 4466 mg GAE/100 g of fresh weight for white mulberry leaves [17]. *M. alba* leaves collected in various regions of Korea were characterized by the content of phenols at the level from 2820 up to 5540 mg GAE/100 g [18]. The authors also showed a strong positive correlation between total phenolic content and DPPH scavenging ability. In our research, the correlation coefficients were above 0.9 (Table 2), which confirms the close relationship between polyphenol content and antioxidant capacity. No correlations were observed between leaf and fruit parameters for the tested cultivars.

Chromatographic analyses of mulberry extracts confirm a much higher content of polyphenol compounds in leaves (2993−8426 mg/kg DW) than fruits (307−1417 mg/kg DW) regardless of the plant variety (Table 3). This is consistent with the results of spectrophotometric measurements of total phenolics content only for white fruits. We speculate that for fruits containing a lot of anthocyanins, the Folin−Ciocalteu method gave false results due to the presence of interfering colored components. The main identified polyphenols were chlorogenic acid isomers and glycosides of quercetin and kaempferol, including numerous acetylated and malonylated derivatives (Table 3). These compounds are the dominant secondary metabolites of mulberry, which was confirmed by literature reports [19,20,21]. However, Yu et al. [8], based on HPLC-MS analysis among 19 tested mulberry cultivars, selected only six that exhibited a high polyphenolics content, resulting in a high antioxidant activity of leaf extracts. Similar to our findings, chlorogenic acid was indicated as the predominant phenolic compound, ranging from 2.45 to 10.24 mg/g DW, while isoquercitrin and rutin were the most abundant flavonoid glycosides (0.70–4.83 and 0.42–4.31 mg/g DW, respectively) in mulberry leaves [8]. For the mulberry leaves, the content of chlorogenic acid ranged from 1.5 to 4.31 mg/g, whereas isoquercitrin and rutin in amounts of 0.09−0.36 and 0.33−0.91 mg/g, respectively, were determined. The differences in the determined levels of the identified compounds may result from the different extraction conditions. In the cited work, extraction with 80% methanol with ultrasound support was used; in our case, the extraction solvent was 50% ethanol.

In our study, cyanidin glycosides were identified in the dark-colored fruit extracts: Cyanidin 3-O-glucoside and cyanidin 3-O-rutinoside. These cyanidin derivatives are the main representatives of this class of compounds found in mulberry pigment, containing cyanidin 3-O-rutinoside (60%) and cyanidin 3-O-glucoside (38%) [22]. The chromatographic method yielded a lower phenolics content for both leaves and fruits (see Table 3). In fruits, the level found by the Folin−Ciocalteu method was 8- to 18-fold higher, which may result from the inaccuracy of the Folin−Ciocalteu method, which is also sensitive to other classes of compounds.

### 2.1. Experiment 1: Addition of Mulberry Leaves and Fruits to Liquefied Honey

Liquefied rape honey was enriched with ground ‘Ukraińska’ and ’Kenmochi’ mulberry leaves and fruits (white and black, respectively) at the final concentration of 4% (*w*/*w*). ’Kenmochi’ mulberry was chosen due to the highest content of polyphenols in black fruits, whereas the ‘Ukraińska’ was selected as a cultivar with slightly pink fruits exhibiting the highest antioxidant activity of leaf extract. Rape honey was used as having the least pro-health properties [23,24], so the addition of plant material should significantly improve its quality. Moreover, this variety crystallizes quickly (within 3 days), which should allow a product of homogenous consistency to be obtained, and to prevent its adverse delamination. Results of the antioxidant and enzymatic activity of prepared honeys are presented in Table 4.

The mulberry enriched honeys exhibited a significantly higher (*p* < 0.05) total phenolics content as compared to raw rape honey. According to various authors, Polish rape honey is relatively poor in phenolic compounds, containing about 4.5 [25] to about 33.5 mg GAE/100 g [26]. The addition of plant material significantly increased the content of polyphenols, and the enrichment ranged from 50 to 140% in comparison to control honey. The increase in polyphenols content was also reflected in the higher antioxidant activity of enriched honeys. Both analytical methods (DPPH and FRAP) used for antioxidant capacity assessment indicated honeys with the addition of ’Ukraińska’ white mulberry leaves as more effective in free radical scavenging and reducing power than those with the addition of ’Kenmochi’ red mulberry. Stronger reducing abilities in FRAP assay were observed in the case of ‘Kenmochi’ red fruits additive, which was associated with a higher content of polyphenols, including anthocyanins in ’Kenmochi’ mulberry red fruits. 

### 2.2. Experiment 2: Addition of Mulberry Leaves and Fruits During Creaming Honey

The technique of producing creamed honey is based on the mechanical stirring of the liquid honey, inoculated with crystal seeds, up to the point of crystallization. Stirring prevents the formation of larger crystals that can occur in unprocessed, naturally crystallized honey. The processing also produces a honey with a smooth spreadable consistency. This technology does not alter the therapeutic and other properties of honey, only slightly modifying the physical properties of the sugar crystals in the honey (Dżugan, unpublished data). However, pro-healthy properties of honey can be strongly reinforced if, during creaming, honey is flavored with herbs or fruits. Thus, creamed honeys with the addition of white mulberry leaves and fruits were prepared. The purpose of this experiment was to obtain the product with homogeneous consistency and check the health-promoting properties of honey enriched during the controlled crystallization process. As the white mulberry is known for its α-amylase and α-glucosidase inhibitory activity, the hypothesis that it will inhibit the activity of native honey glycoside hydrolases has been verified. Table 5 contains data describing polyphenols content, antioxidant capacity, and enzymatic activity (α-glucosidase (α-GLU), β-galactosidase (β-GAL), and diastase (as diastase number)) of the obtained creamed honeys flavored with mulberry plant.

In the creamed honey, we observed an increase in the phenolic compounds as compared to the raw honey. The phenolics content almost quadrupled in the case of 4% added leaves. In the case of the ’Ukraińska’ clone, the enrichment of creamed honey with the leaves brought a better effect than the enrichment with the fruits, which was related to higher contents of polyphenols in the leaves than in the fruits of this clone. Moreover, an upward trend, both in relation to total phenolic content and in antioxidant capacity activity, along with the increasing percentage of the additive were observed. The beneficial modification of the antioxidant properties of honeys creamed with various herbs was previously confirmed [14]. The supplementation with lavender, lemon balm, nettle, peppermint, and ginger resulted in a significant increase in total phenolic content in comparison to multifloral honeys, even up to 10 times (in the case of lemon balm). There was also an increase in the ability of enriched honeys to inhibit the DPPH radical, as well as their reducing power measured as FRAP [14].

For creamed honey samples, the values of the diastase number, which describe the amylolytic activity of honey, were also determined (Table 5). For raw rape honey, the obtained diastase number was at the level of 12.02, which is a relatively low value, but typical for this variety of honey [23,27]. With the increase in the addition of fruit and leaves, i.e., with the increasing concentration of mulberry addition, the diastase number gradually decreased, wherein a statistically significant decrease only for the 4% of additive was observed. It confirmed the inhibitory properties of mulberry leaves and fruits against α-amylase activity − native honey enzyme. Such mulberry inhibitory properties against α-amylase activity were observed before by Habeeb et al. [28] and Nickavar and Mosazadeh [29,30,31]. In the case of α-glucosidase and β-galactosidase, we observed an increase in activity of these enzymes along with the increase in mulberry leaves content in honey. Surprisingly, the addition of fruit caused a slight decrease in activity of α-glucosidase in comparison to the control. The activity of β-galactosidase in honey enriched with fruits was increased, but we did not observe a dependence on the concentration of the addition. The enzymes present in honey can be of both plant and bee origin, although the exact source of particular enzymes is still not explained. It is known that such enzymes as catalase, lysozyme, and acid phosphatase are originated from plants [32]. Thus, the different response of the studied enzymes to mulberry inhibitors may be explainable by their different sensitivity resulting from different origins: From pollen or the hypopharyngeal gland of the honey bee. 

The addition of mulberry to rape honey strongly diversified polyphenolic profiles of the enriched honey, which was determined by ultra-performance liquid chromatography coupled with a mass spectrometer (UPLC-PDA-ESI-MS), and is presented in Table 6.

It is known that the major polyphenolic compounds in honey are phenolic acids and flavonoid aglycones. Tested raw rape honey contained several phenolic compounds in its composition: Mainly hydroxybenzoic acid derivatives and some flavonoid aglycones (quercetin, kaempferol, and pinobanksin). Similarly, Trautvetter et al. [33] found hydroxybenzoic acid derivatives as dominant compounds in rape honey, and also identified apigenin and chrysin among flavonoids. Other authors indicate the presence of taxifolin, tectochtysin, and ellagitannins [34]. In enriched honey samples, we have found many additional polyphenols from leaves and mulberry fruits. They are mainly caffeoylquinic acids (chlorogenic, neocholorogenic, and cryptochlorogenic) and quercetin and kaempferol glycosides. All these compounds have been previously identified in leaf and mulberry extracts. Enrichment with leaves results in the introduction of a 10-fold greater amount of phenolic compounds to honey in comparison to fruits addition (5 and 53 mg/100g, respectively). Comparing to raw honey, the obtained products were strongly reinforced with polyphenols, by 7- and 70-fold, in the case of fruits and leaves, respectively. Although honeys enriched with fruits were preferred by consumers in organoleptic examination, the honeys with the addition of leaves, which exhibited a grey-green color and specific herbal flavor and taste, were also highly evaluated (data not shown).

**Figure 1:**
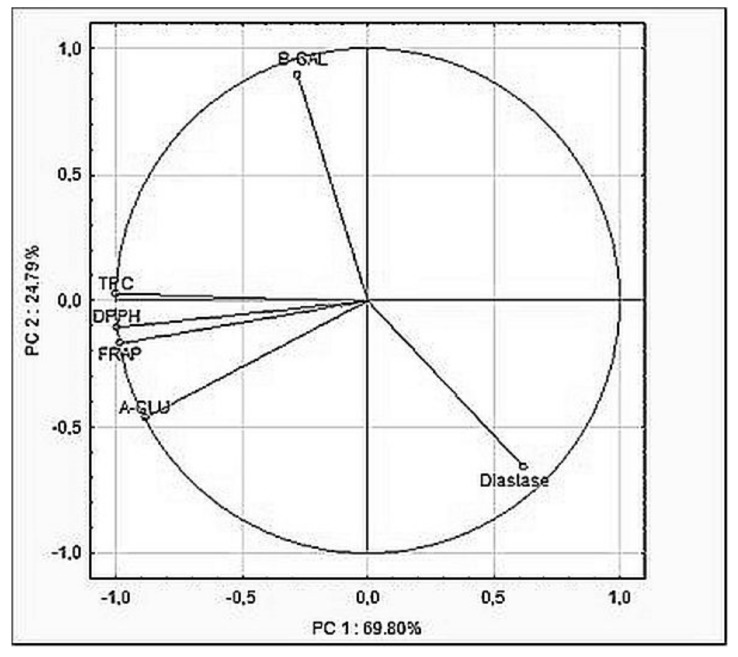
Principal component analysis (PCA)—biplot of scores and loadings of data obtained from antioxidant and enzymatic activity tested for creamed enriched honeys, as well as distribution of variables.

For creamed honey samples, the relation between determined parameters was checked by a principal component analysis (PCA) study (Figure 1). The results indicated the strong positive correlation between TPC, as well as FRAP and DPPH tests (*r* = 0.980 and 0.990, respectively), which indicate a close relationship between the content of phenolic compounds in honey and their antioxidant potential. Moreover, a positive correlation between α-glucosidase activity and antioxidant activity (*r* = 0.870–0.948) was found, whereas a negative correlation between antioxidant activity and diastase activity was calculated (*r* = −0.498–(−0.610)), which indicates that the higher the antioxidant compounds from mulberry, the lower the diastase activity. Moreover, it can be observed that all the tested parameters have a significant impact on the overall quality of honey due to them being located close to the projection circle. PCA analysis has been used in order to evaluate the correlation between the antioxidant capacity and diastase number of various types of honey [35,36]. A positive correlation between these two variables was found. However, in this study, we observed a negative correlation between antioxidant capacity (regardless of the measurement method used) and diastase activity, which support the hypothesis that the addition of mulberry effectively reduces the activity of this enzyme and simultaneously increases antioxidant potential. Results confirming the possibility of the inhibition of α-glucosidase of different origin (microbiological or animal) by mulberry extracts are available in the literature [30,31]. The different situation observed for α-glucosidase and β-galactosidase in the presented study indicates different properties of these enzymes, which are manifested by the lack of sensitivity to plant-derived inhibitors. This may be a consequence of their origin from bee throat glands [37]. However, it is only speculation because the knowledge about the source and function of honey enzymes is still being explored.

## 3. Materials and Methods 

### 3.1. Chemicals

Most of the chemicals and reagents were obtained from Sigma Aldrich (Saint Louis, MO, USA), and buffer components were purchased from POCH (POCH, Gliwice, Poland).

### 3.2. Plants and Honey

Mulberry leaves and fruits (*Morus alba* breeding clones ‘Ukraińska,’ ‘Bistro,’ and ‘Żółwińska wielkolistna,’ and *Morus bombycis* ‘Kenmochi’) were collected in July 2018 in the area of Rzeszów (Rzeszów, Poland, 50°00’N 22°01’E). After botanical identification, done by experts from the Aeropolis Laboratory of Plant Biotechnology (University of Rzeszow, Rzeszów, Poland), the leaves were dried at room temperature, without exposure to sunlight. The fruits were lyophilized using a FreeZone 2.5 lyophilizer (Labconco, Kansas City, MO, USA). Dehydration was carried out for 48 h by heating the shelves to 30 °C at a normal pressure of 0.5 bar. Both leaves and fruits were ground by a laboratory grinder into the form of powder. Voucher specimens of the plant material were deposited in the department archive. For experiments, rape honey from the beekeeping 2018 season obtained from the ecological apiary localized in the Podkarpackie region (J. Bańkowski, Roźwienica, Poland), liquefied at 45 °C during 48 h in a laboratory dryer, was used. 

### 3.3. Plant Extract Preparation for Spectrophotometric Assays

One gram of pulverized dried leaves or freeze-dried fruits was extracted with 20 mL deionized water (5% *w*/*v*) by shaking on the laboratory orbital shaker (MP BT1500, Benchmark, Sayreville, NJ, USA) for an hour at 21 ± 2 °C. Then, the extracts were filtered by filter paper and stored in a freezer at −21 °C for further analysis but no more than 3 months.

### 3.4. Honey Samples Preparation

#### 3.4.1. Experiment 1: Addition of Mulberry Leaves and Fruits to Liquefied Honey

To liquefied rape honey, dried leaves and freeze-dried fruits of two selected mulberry species were added into the concentration of 4% (*w*/*w*). Briefly, honey was weighted into glass jars, an appropriate amount of pulverized dried leaves or freeze-dried fruits was added, and the whole mixture was mixed thoroughly by a kitchen mixer during 60 s. Such prepared samples were stored at 21 ± 2 °C without exposure to sunlight for 30 days until analyses. In the case of delamination of the sample, it was uniformed directly before analyses.

#### 3.4.2. Experiment 2: Addition of Mulberry Leaves and Fruits during Creaming Honey

Liquefied rape honey was inoculated with crystalized honey (99:1 g) and mixed by the kitchen mixer for 60 s four times a day to start the crystallization process. Then, powdered mulberry leaves or fruits were introduced to honey in amounts of 1, 2, and 4% (*w*/*w*), and the whole mixture was mixed again by the kitchen mixer for 60 s. Such prepared samples were stored in a refrigerator at 4 °C for three days and were mixed two times a day for even distribution of additives. After complete crystallization, the samples were stored at 21 ± 2 °C without exposure to sunlight for 30 days until analyses.

### 3.5. Total Phenolics Content Determination

The total phenolics content was determined using a Folin−Ciocalteu reagent, according to Singleton and Rossi [38] with minor modifications. To 0.2 mL of 10% honey solution or plant extract, 1 mL of 10% Folin−Ciocalteu reagent followed by 0.8 mL of 7.5% (*w*/*v*) of Na_2_CO_3_ solution were added. Samples were kept in the dark for 120 min and the absorbance was then measured against blank at 760 nm using a Biomate 3 spectrophotometer (Thermo Scientific, Waltham, MA, USA). The phenolic content expressed as mg of gallic acid (GAE) equivalents per 100 g of honey of plants (mg GAE/100 g) was calculated based on a calibration curve prepared for gallic acid in the range of 0−250 µg/mL (y = 0.0555*x*, *r*^2^ = 0.9976). 

### 3.6. Total Anthocyanins Content Determination

The total anthocyanins in mulberry fruit extracts were determined according to the pH-differential method described by Giusti & Wrolstad [39]. Briefly, the extracts were diluted in two buffers: 0.025 M potassium chloride buffer of pH 1, and 0.4 M acetate buffer of pH 4.5. After 15 min incubation at 21 ± 2 °C, the absorbance was measured at 520 and 700 nm against distilled water using the Biomate 3 spectrophotometer (Thermo Scientific, Waltham, Massachusetts, USA). The absorbance of each sample was calculated using the following formula:

A = (A520 − A700) pH 1.0 − (A520 − A700) pH 4.5.(1)

The total anthocyanins content expressed as cyanidin-3-glucoside equivalents in mg per 100 g of fruits was calculated using the following formula:

TAC = (A × MW × DF × 1000)/(ε × 1),(2)

where:

A—calculated absorbance
MW—molecular weight (449.2 g/mol for cyanidin 3-glucoside)
DF—dilution factor (20)
ε—molar absorptivity of cyanidin 3-glucoside (26900 dm^3^/mol × cm)

### 3.7. Polyphenolic Profile UPLC Analysis

#### 3.7.1. Sample Preparation

The plant sample extract was prepared by grinding 1 g of the samples (dried leaves or lyophilized fruits) in a mortar with 20 mL of solvent (50% ethanol, POCH, Gliwice, Poland), and was placed for 15 min in an ultrasonic water bath (Polsonic, SONIC 10, Poland) without additional heating. Next, samples were filtered through filter paper and directly filtered through 0.20 μm nylon syringe filters before analysis. 

Honey sample extract was obtained by dissolving 30 g of honey in 100 mL of acidified water (pH 2). The solution was filtered through a 0.20 µm nylon filter disc and passed through a Sep Pak C18 Cartridge (Waters, Milford, CT, USA) preconditioned with acidified water and methanol and subsequently rinsed with 10 mL of acidified water. The phenolic compounds remained on the column, while sugars and other polar compounds were eluted with the aqueous solvent. The whole phenolic fraction was then eluted with 10 mL of HPLC-grade methanol, and the solvent was evaporated in a rotary evaporator Hei-VAP Advantage (Heidolph Instruments, Walpersdorfer, Schwabach, Germany) at 40 °C under reduced pressure. The residue was redissolved in 1 mL of a 50:50 (*v*:*v*) HPLC-grade acetonitrile and water mixture. Finally, all extracts were filtered through a 0.20 µm nylon filter disc and subjected to UPLC–MS analysis (Waters, Manchester, Great Britain).

#### 3.7.2. UPLC Separation

Testing of the polyphenolic profile of dried leaves and lyophilized fruits was performed by ultra-performance liquid chromatography coupled with a mass spectrometer (UPLC-PDA-ESI-MS, Waters, Manchester, Great Britain). For this purpose, an Aquity ultra-performance liquid chromatograph from Waters (Micromass, Manchester, Great Britain) equipped with a diode array detector (PDA) and a tandem mass detector in the form of a double quadrupole (TQD) was used. The separation was carried out on a C18 BEH column with dimensions of 100 mm × 2.1 mm and a grain size of 1.7 μm (Waters). The following gradient mobile phase was applied: From 5% B (40% acetonitrile) and 95% A (0.1% of an aqueous formic acid solution) to 100% B and 0% A in 8 min. The separation was performed at a mobile phase speed of 0.35 mL/min and a column temperature of 50 °C. The analysis time was 9.5 min. The injection volume was 5 μL. The parameters of the mass detector were as follows: 3.5 kV capillary voltage, 45 V sample voltage, and the temperatures of the ion source and desolvation were 120 °C and 350 °C, respectively. Nitrogen with a flow rate of 800 L/h was used as the carrier gas. The detection was carried out in the negative ion mode in the *m*/*z* range from 120 to 1200. Data collection and analysis were performed using Mass-Lynx 4.1 software (Waters).

The runs of polyphenolic compounds were monitored at the following wavelengths: Phenolic acids at 320 nm, flavonol glycosides at 350 nm, and anthocyanins at 520 nm. Characterization of the single components was carried out via retention time (Rt), spectra, accurate molecular masses, literature data, and pure standards, if available. Calibration curves at concentrations ranging from 0.05 to 5 mg/mL were made for chlorogenic acid, ferulic acid, quercetin-3-O-rutinoside, kaempferol 3-O-glucoside, and cyanidin-3-O-glucoside.

#### 3.7.3. Method Validation

The method was validated for parameters such as linearity, accuracy (relative error, RE), limit of detection (LOD), limit of quantification (LOQ), and precision (relative standard deviation, RSD). Stock standard solutions of the five polyphenols (chlorogenic acid, ferulic acid, quercetin-3-*O*-rutinoside, kaempferol 3-*O*-glucoside, and cyanidin-3-*O*-glucoside) were prepared with methanol. Six calibrators of each standard were prepared by dilution of stock solutions, and the calibration curve was generated by plotting the peak area ratio of the polyphenol versus the nominal concentration. The regression equation was obtained by weighted (1/c2) least-squares linear regression. The LOD was determined as a signal-to-noise ratio (S/N) of 3:1, and the LOQ was determined as a S/N of >10. An acceptable RE within ±20% and an RSD not exceeding 20% should be obtained.

The regression equations were y = 1.66*x* + 0.0892; y = 0.029*x* + 0.028; y = 0.073*x* + 0.0616; y = 0.0622*x* − 0.0004; and y = 0.0373*x* + 0.0128, respectively. The coefficients of determination (R2) were not lower than 0.998. The LODs were 0.029, 0.220, 0.074, 0.049, and 0.186 μg/mL, and the LOQs were 0.0965, 1.2, 3.712, 0.1992, and 0.97 μg/mL, with an RSD and RE within 20% verified by repeated analysis.

### 3.8. Antioxidant Assays

#### 3.8.1. DPPH Test

DPPH (2,2-diphenyl-1-picrylhydrazyl) radical inhibition was measured according to the assay described by Blois [40] with minor modifications. The aliquot of 0.2 mL of 20% honey solution or plant extract was mixed with 1.8 mL of DPPH radical methanolic solution (0.1 mM) and kept in the dark for 60 min. After incubation, the absorbance of the tested and control samples was measured at 517 nm against methanol. The reduction of DPPH radicals was calculated using the following equation:

AA% = [(A0 − As)/A0] × 100,(3)

where A0 is the absorbance of control and As is the absorbance of the tested samples.

#### 3.8.2. FRAP Assay

The FRAP assay (ferric reducing ability of plasma) was carried out according to Bertoncelj et al. [41]. The FRAP reagent contained 2.5 mL of a 10 mM 2,4,6-tripyridyltriazine (TPTZ) solution in 40 mM HCl, 2.5 mL of 20 mM FeCl_3_, and 25 mL of 0.3 M acetate buffer (pH 3.6). Briefly, to 0.2 mL of honey or plant extract, 1.8 mL FRAP reagent was added, and the absorbance of the reaction mixture was measured spectrophotometrically (Biomate 3 spectrophotometer, Thermo Scientific, Waltham, MA, USA) against blank at 593 nm after incubation at 37 °C during 10 min. The results were expressed as μmol of Trolox (TE) equivalents per gram of honey or plants (mmol/g) based on the calibration curve (y = 0.026*x*, *R*^2^ = 0.998), prepared for 0.1 mmol Trolox in the range of 15–200 nmol.

### 3.9. Enzymatic Activity Assays

The activity of two glycolytic enzymes: α-glucosidase and β-galactosidase, using the method described by Dżugan et al. [42] was tested for honey samples. The reaction mixture contained 25 μL of 2 mM appropriate p-nitrophenyl substrate (Sigma Aldrich, USA) in 0.2 M citrate buffer (pH 5.5 for α-GLU and 4.0 for β-GAL) and 25 μL of honey aqueous solution (20% *w*/*w*) sample. In blank, 25 μL of suitable buffer was used instead of substrate solution. Samples were incubated at 37 °C for 90 min, and the reaction was then stopped by the addition of 250 μL of 0.5 M carbonate buffer at pH 10.5. Absorbance was measured on an ELISA reader (Clindiag Systems, Belgium) at a wavelength of 400 nm. Results were expressed as enzymatic units U/kg (μmol/min/kg) by multiplying the measured absorbance by a factor of 52.91. 

### 3.10. Diastase Number Determination

Determination of the diastatic activity of honey was performed using the Phadebas Honey Diastase Test (© Magle AB Lund, Sweden) according to the procedure attached to the test kit. An aliquot of 5 mL of 1% honey solution in 0.1 M acetate buffer (pH 5.2) was transferred to a test tube and incubated in 40 °C for 5 min. Next, a Phadebas tablet was added and, after 30 min incubation at 40 °C, the reaction was terminated by adding 1 mL of 0.5 M NaOH. The samples were then filtered through filter paper and the absorbance was measured at 620 nm using a Biomate 3 spectrophotometer (Thermo Scientific, Waltham, MA, USA). The results were read according the table appended to the test kit and were expressed as DN.

### 3.11. Statistical Analysis

All calculations were done in three replications. For obtained data, the means and standard deviations were calculated. The correlations among obtained parameters were analyzed by the Pearson coefficient (*r*). The significant differences were calculated by a one-way analysis of variance, followed by Tukey’s honest significant difference test (*p* < 0.05). Principal component analysis (PCA) was applied to find the relation between the tested parameters in honey enriched in leaves and fruits during creaming. All calculations were made using the Statistica 13.1 software (StatSoft, Tulsa, OK, USA).

## 4. Conclusions

Leaves and fruits of white mulberry (*Morus alba*) can be used as an admixture to creamed honeys, in order to enrich them in polyphenol compounds and improve their pro-health properties. Naturally poor in polyphenols, rape honey has been significantly enriched with numerous flavonoids and phenolic acids, and its antioxidant activity has been repeatedly increased. The reinforcing effect was species- and cultivar-dependent, and the better enrichment of honey was achieved with the use of mulberry leaves than fruits. The significant effect of the addition of mulberry on the quality and properties of honey product was confirmed with PCA analysis. It has been shown that the native honey diastase activity was negatively impacted by the increasing mulberry leaves admixture while an opposite tendency for α-glucosidase was observed. 

## Figures and Tables

**Table 1:** Total phenolics content (TPC), radical scavenging activity (2,2-diphenyl-1-picrylhydrazyl (DPPH) test), reducing antioxidant powers (ferric reducing ability of plasma (FRAP) assay) for leaves and fruits, and total anthocyanins content (TAC) for fruits of tested mulberry cultivars expressed per dry weight.

Sample		TPC (mg GAE/100 g)	DPPH (% of Inhibition)	FRAP (mmol TE/100 g)	TAC (mg/100 g as cyanidin 3-glucoside)
*M. alba* ‘Bistro’	leaves	572.8 ± 40.9 ^A^	22.2 ± 1.1 ^A^	1.58 ± 0.07 ^A^	-
	fruits (white)	426.7 ± 25.2 ^a^	16.3 ± 2.2 ^a^	1.28 ± 0.05 ^a^	76.3 ± 8.6 ^a^
*M. alba* ‘Żółwińska wielkolistna’	leaves	680.6 ± 58.9 ^AB^	18.7 ± 1.0 ^A^	1.97 ± 0.08 ^B^	-
	fruits (black)	1041.1 ± 56.7 ^bc^	78.9 ± 1.5 ^b^	5.43 ± 0.12 ^b^	618.2 ± 130.8 ^b^
*M. alba* ‘Ukraińska’	leaves	761.4 ± 56.2 ^B^	63.5 ± 2.9 ^B^	3.02 ± 0.22 ^C^	-
	fruits (slightly pink)	302.5 ± 10.8 ^a^	5.3 ± 2.8 ^c^	0.86 ± 0.02 ^c^	61.8 ± 13.1 ^a^
*M. bombycis* ‘Kenmochi’	leaves	665.5 ± 63.3 ^C^	54.6 ± 2.8 ^C^	2.15 ± 0.08 ^B^	-
	fruits (black)	1114.8 ± 86.6 ^AB^	77.9 ± 1.6 ^b^	5.20 ± 0.06 ^d^	763.5 ± 86.0 ^b^

Data as mean value ± standard deviation (SD; *n* = 3). ^a,b,c,d^—Means for fruits marked with the different superscript letter within the column are significantly different (Tukey’s honest significant difference test, *p* < 0.05). ^A,B,C^—Means for leaves marked with the different superscript letter within the column are significantly different (Tukey’s honest significant difference test, *p* < 0.05).

**Table 2:** Correlation matrix for mulberry fruits and leaves.

		TPC		DPPH		FRAP	
		Fruits	Leaves	Fruits	Leaves	Fruits	Leaves
TPC	Fruits	1.000					
	Leaves	−0.162	1.000				
DPPH	Fruits	0.997 *	−0.147	1.000			
	Leaves	−0.201	0.689 *	−0.250	1.000		
FRAP	Fruits	0.992 *	−0.100	0.999 *	−0.236	1.000	
	Leaves	−0.337	0.959 *	−0.340	0.832 *	−0.301	1.000
TAC	Fruits	0.990 *	-	0.980 *	-	0.978 *	-
	Leaves	-	-	-	-	-	-

*—Correlation coefficient statistically significant (*p* < 0.05).

**Table 3:** Individual phenolic compounds (mg/kg) identified by ultra-performance liquid chromatography coupled with a mass spectrometer (UPLC-PDA-ESI-MS) in mulberry leaves and fruits.

Compound		Rt	λ_max_	[M − H]^−^ *m*/*z*		*M. alba* ‘Bistro’		*M. alba* ’Żółwińska wielkolistna’		*M. alba* ‘Ukraińska’		*M. bombycis* ‘Kenmochi’	
		min.	nm	MS	MS/MS	Leaves	Fruits (White)	Leaves	Fruits (Black)	Leaves	Fruits (Pink)	Leaves	Fruits (Black)
1	Neochlorogenic acid	2.51	299sh, 327	353	191, 179	260.6	18.0	113.4	18.0	93.9	22.1	174.3	57.2
2	Cyanidin 3-*O*-glucoside	2.98	279, 515	449^*^	287	ND	Tr	ND	119.6	ND	ND	ND	239.2
3	Cyanidin 3-*O*-rutinoside	3.08	279, 515	595^*^	287	ND	ND	ND	123.2	ND	ND	ND	246.4
4	Chlorogenic acid	3.12	299sh, 325	353	191, 179	4317.0	18.1	1549.2	104.5	1498.9	25.6	2182.1	315.4
5	Cryptochlorogenic acid	3.25	299sh, 327	353	191, 179	1217.7	19.6	437.1	77.9	340.5	21.4	787.6	238.4
6	Quercetin 3-*O*-[(6”-*O*-malonyl)-glucosyl]-glucoside	3.36	255, 345	711	625, 301	47.7	12.9	8.0	47.3	49.6	13.8	30.2	68.6
7	Kaempferol 3-*O*-rutinoside-7-*O*-glucoside	3.47	264, 345	755	593, 285	21.2	18.7	14.6	Tr	10.0	18.9	0.7	Tr
8	Caffeoylquinic acid	3.72	299sh, 327	355	191	108.6	17.6	24.0	Tr	54.5	17.0	71.5	Tr
9	Kaempferol 3-*O*-glucosyl-glucoside-7-*O*-glucoside	3.80	266, 312	755	609, 285	42.2	17.1	16.8	Tr	02.8	17.8	40.1	Tr
10	Quercetin 3-*O*-glucosyl-glucoside	4.05	254, 347	625	301	76.7	9.1	44.5	Tr	ND	8.0	44.1	0.8
11	Quercetin 3-*O*-rutinoside-7-*O*-rhamnoside	4.14	255, 350	755	609, 301	12.8	7.6	22.6	Tr	42.9	7.6	96.2	Tr
12	Quercetin 3-*O*-rhamnosyl-glucoside	4.45	254, 347	609	301	06.8	7.6	22.4	Tr	06.8	7.6	58.0	Tr
13	Kaempferol 3-*O*-rutinoside-7-*O*-rhamnoside	4.52	264, 338	739	593, 285	3.3	17.3	Tr	Tr	Tr	17.1	14.2	Tr
14	Quercetin 3-*O*-rutinoside	4.67	255, 354	609	301	908.9	10.6	329.3	70.7	450.3	12.1	557.5	188.9
15	Quercetin 3-*O*-[(6”-*O*-malonyl)-glucosyl]-rhamnoside	4.82	255, 352	695	609, 301	58.0	10.0	19.6	ND	46.2	08.8	208.3	ND
16	Quercetin 3-*O*-glucoside	4.89	255, 355	463	301	200.6	14.5	90.7	7.0	200.2	13.2	363.9	48.6
17	Quercetin 3-*O*-(6”-acetyl)-glucoside	5.15	255, 354	505	463, 301	726.9	12.8	180.4	Tr	469.6	09.9	1062.6	13.6
18	Kaempferol 3-*O*-rutinoside	5.19	264, 347	593	285	79.9	17.9	31.4	Tr	43.0	17.6	102.2	Tr
19	Kaempferol 3-*O*-[(6”-malonyl)-glucosyl]-rhamnoside	5.28	264, 347	679	593, 285	3.4	17.1	Tr	Tr	Tr	17.1	56.8	Tr
20	Quercetin 3-*O*-rhamnoside	5.39	255, 354	447	301	174.9	10.1	31.7	Tr	130.5	09.0	234.0	Tr
21	Quercetin 3-*O*-(6”-malonyl)-glucoside	5.79	264, 347	533	447, 285	152.7	19.5	35.5	Tr	125.7	17.8	452.1	Tr
22	Ferulic acid	6.27	299sh, 327	193	179, 134	Tr	15.6	Tr	0.03	Tr	17.6	Tr	Tr
23	Quercetin	6.99	255, 355	301	-	6.4	7.6	21.9	Tr	Tr	07.6	Tr	Tr
**Total**						**8426.4**	**299.1**	**2993.2**	**56.84**	**3565.1**	**307.5**	**6536.3**	**1417.1**

*—*m*/*z* for anthocyanins have been obtained in the positive mode [M + H]^+^, Tr—traces (below limit of detection), ND—not detected.

**Table 4:** Total phenolic compounds content (TPC), radical scavenging activity (DPPH test), and reducing antioxidant powers (FRAP assay) in mulberry enriched honeys (4% *w*/*w*).

Sample	TPC (mg GAE/100 g)	DPPH (% of Inhibition)	FRAP (mmol TE/g)
Honey (Control)	25.2 ± 1.2 ^a^	2.9 ± 0.1 ^a^	0.035 ± 0.002 ^a^
Honey + *M. alba* ‘Ukraińska’ fruits	38.0 ± 1.1 ^b^	23.6 ± 2.7 ^b^	0.080 ± 0.006 ^b^
Honey + *M. alba* ‘Ukraińska’ leaves	51.8 ± 1.5 ^c^	32.1 ± 1.1 ^c^	0.217 ± 0.003 ^c^
Honey + *M. bombycis* ‘Kenmochi’ fruits	59.7 ± 2.0 ^d^	27.2 ± 1.9 ^b^	0.269 ± 0.011 ^d^
Honey + *M. bombycis* ‘Kenmochi’ leaves	50.8 ± 5.5 ^c^	22.9 ± 1.9 ^b^	0.186 ± 0.003 ^e^

Data as mean value ± standard deviation (SD; *n* = 3). ^a,b,c,d,e^—Means marked with different superscript letter within the column are significantly different (Tukey’s honest significant difference test, *p* < 0.05).

**Table 5:** Total phenolic compounds content (TPC), radical scavenging activity (DPPH test), reducing antioxidant powers (FRAP assay), and activity of α-glucosidase (α-GLU) and β-galactosidase (β-GAL), as well as diastase number, in tested enriched creamed honeys.

Sample	TPC (mg GAE/kg)	DPPH (% of inhibition)	FRAP (μmol TE/g)	α-GLU Activity (U/kg)	β-GAL Activity (U/kg)	Diastase Number
Control	214.1 ± 15.4 ^a^	3.0 ± 0.4 ^a^	64.2 ± 4.4 ^a^	5.7 ± 0.0 ^a^	17.0 ± 1.8 ^a^	12.0 ± 2.5 ^a^
Leaves 1%	314.7 ± 10.7 ^b^	11.2 ± 1.5 ^b^	118.3 ± 6.8 ^b^	6.0 ± 0.2 ^a^	17.0 ± 1.7 ^a^	9.7 ± 1.3 ^ab^
Leaves 2%	526.4 ± 35.2 ^c^	27.8 ± 2.6 ^c^	226.0 ± 9.1 ^c^	7.2 ± 0.2 ^b^	23.4 ± 0.3 ^b^	9.3 ± 1.0 ^ab^
Leaves 4%	786.8 ± 1.0 ^d^	55.0 ± 2.3 ^d^	369.7 ± 6.0 ^d^	9.5 ± 0.8 ^c^	26.1 ± 0.3 ^bcd^	7.5 ± 0.8 ^b^
Fruits 1%	294.9 ± 7.3 ^be^	3.7 ± 1.2 ^a^	71.5 ± 2.7 ^ae^	4.3 ± 0.5 ^d^	29.4 ± 2.3 ^cd^	9.9 ± 0.9 ^ab^
Fruits 2%	342.3 ± 11.3 ^bf^	9.1 ± 1.0 ^be^	85.5 ± 2.4 ^e^	4.4 ± 0.2 ^de^	27.6 ± 0.2 ^d^	8.7 ± 0.6 ^ab^
Fruits 4%	385.0 ± 7.3 ^g^	13.5 ± 0.8 ^bf^	107.4 ± 3.4 ^b^	4.9 ± 0.4 ^adf^	26.6 ± 0.7 ^d^	6.8 ± 0.5 ^b^

Data as mean value ± standard deviation (SD; *n* = 3). ^a,b,c,d,e,f,g^—Means marked with different superscript letter within the column are significantly different (Tukey’s honest significant difference test, *p* < 0.05).

**Table 6:** Effect of addition of leaves or fruits mulberry (1% *w*/*v*) on the phenolic profile of rape honey.

Compound		Rt	[M − H]^−^ *m*/*z*		Rape Honey (mg/kg)	
		min.	MS	MS/MS		
1	Gallic acid	1.04	169	125	0.045	
2	Caffeic acid	3.99	179	135	0.068	
3	coumaric acid	4.28	163	119	0.032	
4	3-hydroxy benzoic acid	4.3	137	93	0.180	
5	4-hydroxy benzoic acid	4.67	137	93	0.008	
6	Syringic acid	4.87	197	140	0.104	
7	Sinapic acid	4.89	223	175	0.074	
8	Quercetin	7	301		0.013	
9	Kaempferol	8.16	285		0.168	
10	Pinobanksin	8.24	271		0.002	
**Total**					**0.694**	
**Enriched honey**					**+ 1% (*w*/*w*) Fruits** **(mg/kg)**	**+ 1% (*w*/*w*) Leaves** **(mg/kg)**
1	Neochlorogenic acid	2.51	353	191, 179	0.048	Tr
4	Chlorogenic acid	3.12	353	191, 179	1.503	16.341
5	Cryptochlorogenic acid	3.25	353	191, 179	0.832	2.371
6	Quercetin 3-*O*-[(6”-*O*-malonyl)-glucosyl]-glucoside	3.36	711	625, 301	0.020	0.944
7	Kaempferol 3-*O*-rutinoside-7-*O*-glucoside	3.47	755	593, 285	0.006	0.650
8	Caffeoylquinic acid	3.72	355	191	0.086	1.209
9	Kaempferol 3-*O*-glucosyl-glucoside-7-*O*-glucoside	3.80	755	609, 285	Tr	0.256
10	Quercetin 3-*O*-glucosyl-glucoside	4.05	625	301	0.239	0.234
11	Quercetin 3-*O*-rutinoside-7-*O*-rhamnoside	4.14	755	609, 301	0.071	1.173
12	Quercetin 3-*O*-rhamnosyl-glucoside	4.45	609	301	0.138	1.033
13	Kaempferol 3-*O*-rutinoside-7-*O*-rhamnoside	4.52	739	593, 285	0.310	0.456
14	Quercetin 3-*O*-rutinoside	4.67	609	301	0.981	6.972
15	Quercetin 3-*O*-[(6”-*O*-malonyl)-glucosyl]-rhamnoside	4.82	695	609, 301	0.229	0.967
16	Quercetin 3-*O*-glucoside	4.89	463	301	0.282	8.556
17	Quercetin 3-*O*-(6”-acetyl)-glucoside	5.15	505	463, 301	0.024	6.667
18	Kaempferol 3-*O*-rutinoside	5.19	593	285	0.089	1.673
19	Kaempferol 3-*O*-[(6”-malonyl)-glucosyl]-rhamnoside	5.28	679	593, 285	0.077	0.187
20	Quercetin 3-*O*-rhamnoside	5.39	447	301	0.026	1.784
21	Quercetin 3-*O*-(6”-malonyl)-glucoside	5.79	533	447, 285	0.030	1.580
22	Ferulic acid	6.27	193	179, 134	Tr	Tr
23	Quercetin	6.99	301	-	0.033	0.030
**Total**					**5.024**	**53.084**

Tr—traces (below limit of detection).

## Abbreviations

DN: diastase number; DNJ, 1-deoxynojirimycin; α-GLU, α-glucosidase; β-GAL, β-galactosidase
